# Dr. Bache, on a Successful Case of Asthma

**Published:** 1799-09

**Authors:** W. Bache

**Affiliations:** Birmingham


### *To the Conductors of the Medical and Phyfical Journal.*

GENTLEMEN,

AS I have been requefted for a ftatement of an *afthmatic cafe* that came under my care fome time ago, I have referred to my memorandums refpecting it; and as I have ftated the particulars of it, if you think the perufal of them may prove agreeable to any of the readers of your valuable publication, I fubmit them to your difpofal.

I am, refpectfully,

BIRMINGHAM, Your moft humble Servant,

*July* 15*th*, 1799. W. BACHE.

Mr. PARKER, an attorney of eminence and refpectability in Birmingham, when I was firft called to him had been for feveral years feverely afflicted by afthma, that reduced him to a ftate of extreme weaknefs and emaciation. He had previoufly fubjected himfelf to the medical advice of feveral phyficians of well-eftablifhed reputation, and had at times received fome temporary relief.

Several weeks before I faw him, he had been removed from his houfe in town to a fine dry fpot, about a mile diftant from Birmingham, for the benefit of the air, but had not experienced any fenfible advantage from the change of fituation; and feveral of his friends and acquaintance freely declared to me, that they thought the violence of his complaint was fuch, as muft neceffarily terminate his exiftence in a few days.

Upon enquiry, I found he had experienced a confiderable degree of dyfpepfia, and that it had attended him almoft conftantly for feveral years, but the period of its origin was not within the compafs of his recollection. I could not difcover any inflammatory affection in the fyftem, either general or local; flight fpafmodic and convulfive ones had frequently exerted an influence upon various parts of the body. His fleep alfo was frequently broken, and much difturbed by unpleafant dreams. He was remarkably fubject to changes, both in his fenfations and animal fpirits, from the viciffitudes of the atmofphere.

His faliva was remarkably frothy, and his expectoration extremely vifcid. The alvine fæces oft-times affumed the appearance of yeaft in a ftate of effervefcence. The urine was in common of a natural colour; when it had ftood a few hours an eneorema generally appeared in it, and *its furface feemed as if covered with duft.* Being ftruck by this uncommon appearance, to afcertain its nature, I bent a ftrip of writing paper about three inches long long, and half that width, in its middle (to a right angle), I then laid one of its ſquare ſurfaces flat upon the ſurfaces of the urine in the glaſs, and upon raiſing it perpendicularly, found the matter attached to it; I dried it gently, and then ſubjected it to examination by the microſcope, which convinced me that what I had miſtaken for duſt were ſaline cryſtals, but I was unable to collect a ſufficiency of them for any chemical experiment.

When I poured a ſmall quantity of very tranſparent lime-water into Mr. Parker's urine, the lime contained in it was always precipitated by it with more celerity, than when I applied the ſame calcarious ſolution, in like manner and proportion, to my own.

The matter he perſpired generally ſmelt ſour; and when pieces of bibulous paper were previouſly ſtained by a ſolution of litmus, and then applied to various parts of the body, the exudation from each produced the effect of a weak acid upon the colour; and when the paper was made dry, and the edge of it applied to the flame of a candle, I found it a weak touch-paper, but when I diſſolved about a grain or two of vegetable alkali, in a ſmall wine-glaſs of water, immerſed the paper in that, and dried it a ſecond time, I always found that its property as a touch-paper was greatly augmented.

From theſe obſervations I became convinced, that an acid pervaded the whole of the circulating ſyſtem, and I preſumed that it exiſted in a morbid degree, either as to quantity or ſtrength, and was the exciting cauſe of the ſpaſmodic affections obſervable in the lungs, and other membranous parts, to which it might occaſionally be applied, probably ſometimes in a gaſeous ſtate, and at others in a more denſe and concentrated one, and perhaps variouſly combined. The indications of cure ſuggeſted to my mind, were to reſtrain its influence, by the application of alkalies, cretaceous and abſorbent earths; my choice of each was governed by the exiſting circumſtances, at the time when each was preſcribed, and my attention was principally directed to the ſtate of the ſtomach, the bowels, the expectorations, the kidneys and the ſkin.

Gum guaiac. bitters, aromatics, and eſſential oils, I ordered occaſionally, as I wiſhed an increaſed, or otherwiſe diverſified action in different parts of the ſyſtem; and when an augmentation of ſpaſmodic affections took place, or coma, vigil, or dryneſs of the ſkin, I had occaſional reſource both to opium and camphor, which were not only internally, but externally applied, combined with ſolutions of volatile alkali.

Theſe were the whole of the medical means I employed; but as I believe believe that the neglect of proper attention to prophylactics, and improprieties in cloathing, are the primary caufe and chief fupport of the majority of chronic complaints, to prevent a relapfe previous to a confirmed ftate of the functions of his ftomach, &c. I thought proper to recommend a clofe attention to articles of diet, moderately warm cloathing, and rather fparing meals. The importance of proper maftication I alfo endeavoured to point out, and ferioufly urged an attention to it. Animal food I defired might be his principal fubfiftence. I was much pleafed to find every particular of diet was cautioufly attended to, as a complete cure was finally accomplifhed, and it has continued to the prefent time.

